# Synthesis and Anticancer Activity of 4*β*-Triazole-podophyllotoxin Glycosides

**DOI:** 10.1007/s13659-015-0057-3

**Published:** 2015-04-14

**Authors:** Cheng-Ting Zi, Gen-Tao Li, Yan Li, Jun Zhou, Zhong-Tao Ding, Zi-Hua Jiang, Jiang-Miao Hu

**Affiliations:** State Key Laboratory of Phytochemistry and Plant Resources in West China, Kunming Institute of Botany, Chinese Academy of Sciences, Kunming, 650201 China; Key Laboratory of Medicinal Chemistry for Nature Resource, Ministry of Education, School of Chemical Science and Technology, Yunnan University, Kunming, 650091 China; Department of Chemistry, Lakehead University, 955 Oliver Road, Thunder Bay, ON P7B 5E1 Canada

**Keywords:** Podophyllotoxin, 4*β*-Triazole-podophyllotoxin, Glycosides, Click reaction, Anticancer, Synthesis

## Abstract

A series of novel 4*β*-triazole-podophyllotoxin glycosides were synthesized by utilizing the Click reaction. Evaluation of cytotoxicity against a panel of five human cancer cell lines (HL-60, SMMC-7721, A-549, MCF-7, SW480) using MTT assay shows that most of these compounds show weak cytotoxicity. It was observed that compound **16** shows the highest activity with IC_50_ values ranging from 2.85 to 7.28 μM, which is more potent than the control drugs etoposide and cisplatin against four of five cancer cell lines tested. Compound **16** is characterized with an *α*-d-galactosyl residue directly linked to the triazole ring and a 4′-OH group on the E ring of the podophyllotoxin scaffold. HPLC investigation of representative compound indicates that incorporation of a sugar moiety seems to improve the chemical stability of the podophyllotoxin scaffold.

## Introduction

**Fig. 1:**
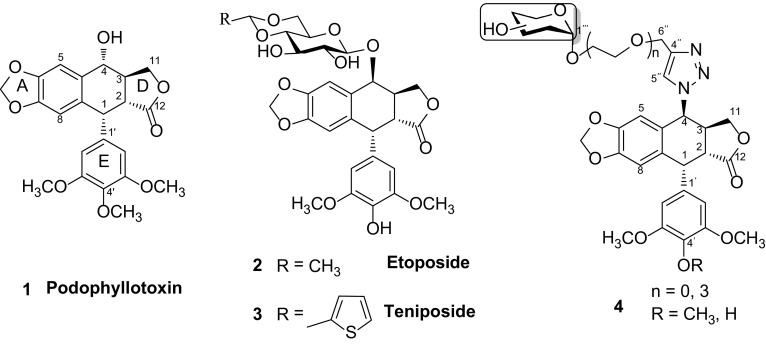
Structures of podophyllotoxin **1**, etoposide **2**, teniposide **3**, and designed podophyllotoxin glycoconjugates **4**

Podophyllotoxin (**1**, Fig. 1), a naturally occurring cyclolignan, which is mainly isolated from the roots of *Podophyllotoxin peltatum*, shows strong cytotoxic activity against various cancer cell lines by inhibiting tubulin through binding with part of its colchicine domain [1, 2]. Due to its severe side effects, podophyllotoxin has limited applications as a drug in cancer chemotherapy, but its semisynthetic derivatives etoposide **2** and teniposide **3** (Fig. 1) are in clinical use for the treatment of a variety of malignancies, including small cell lung cancer, testicular carcinoma, lymphoma, and Kaposi’s sarcoma [3, 4]. However, their therapeutic uses are often hindered by problems such as acquired drug-resistance and poor water solubility. Numerous studies [5–7] have shown that 4*β*-N-substituted derivatives of podophyllotoxin maintain the anticancer activity and function as topoisomerase II inhibitors. Since 1,2,3-triazole ring is a widespread functional group in drug [8], it is intriguing to attach 1,2,3-triazoles to podophyllotoxin derivatives.

Previously Reddy et al. [9] reported several glycosylated 4*β*-triazole-podophyllotoxin derivatives and their anticancer activity. In our earlier study [10], we reported a group of 4*β*-triazole-linked glucose podophyllotoxin conjugates as a new class of antitumor compounds. Reported here are the chemical synthesis of a series of 4*β*-triazole-podophyllotoxin α*-*glycosides (**4**, Fig. 1) of d-galactose, d-mannose, or d-xylose, and their in vitro anticancer activity against five human cancer cell lines, including HL-60 (leukemia), SMMC-7721 (hepatoma), A-549 (lung cancer), MCF-7 (breast cancer), and SW480 (colon cancer).

## Results and Discussion

### Chemical Synthesis

**Scheme 1:**
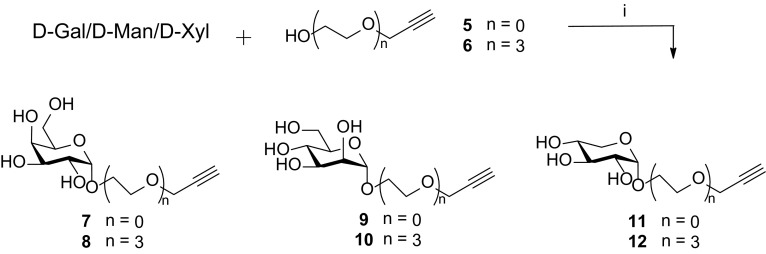
Reagents and reaction conditions: (**i**) cat. H_2_SO_4_–silica, 65 °C, 19–35 %

The Click reaction is a powerful means for linking two units, one with an azide functionality and the other an alkyne functional group. Typically, the cycloaddition reaction mediated by Cu(I) as the catalyst leads to the generation of a 1,4-disubstituted 1,2,3-triazole ring [11, 12]. The preparation of terminal alkynes is shown in Scheme 1. Using sulfuric acid on silica (H_2_SO_4_–silica) catalyzed Fischer type glycosylation with various alcohols and free sugars [13–15], *α*-glycosides with a propargyl group (**7**–**12**) were all obtained. The preparation of compounds **7** [16], **8** [17], **9** [18], **10** [19], and **11** [20] has been reported in the literature.

**Scheme 2:**
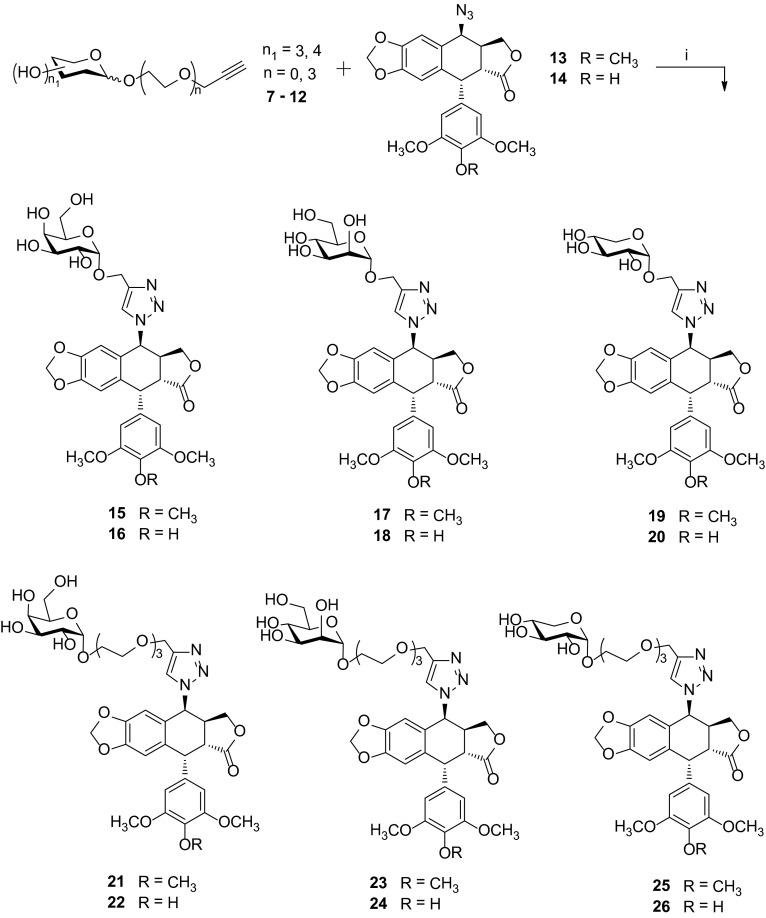
Reagents and reaction conditions: (**i**) CuSO_4_·5H_2_O, sodium ascorbate, *t*-BuOH:H_2_O (1:2), 4 h, rt. 75–87 %

To introduce the azido functionality for the Click reaction, podophyllotoxin was readily converted to 4*β*-azido-4-deoxypodophyllotoxin **13** and 4*β*-azido-4-deoxy-4′-demethylpodophyllotoxin **14** according to previous reports [21, 22]. The azides **13** and **14** were to react with the terminal-alkynes **7**–**12** in the presence of copper (II) acetate and sodium ascorbate in *t*-butyl alcohol and water (1:2) at room temperature for 4 h to provide 4*β*-triazole-podophyllotoxin glycosides **15**–**26** in good yield (Scheme 2).

**Fig. 2:**
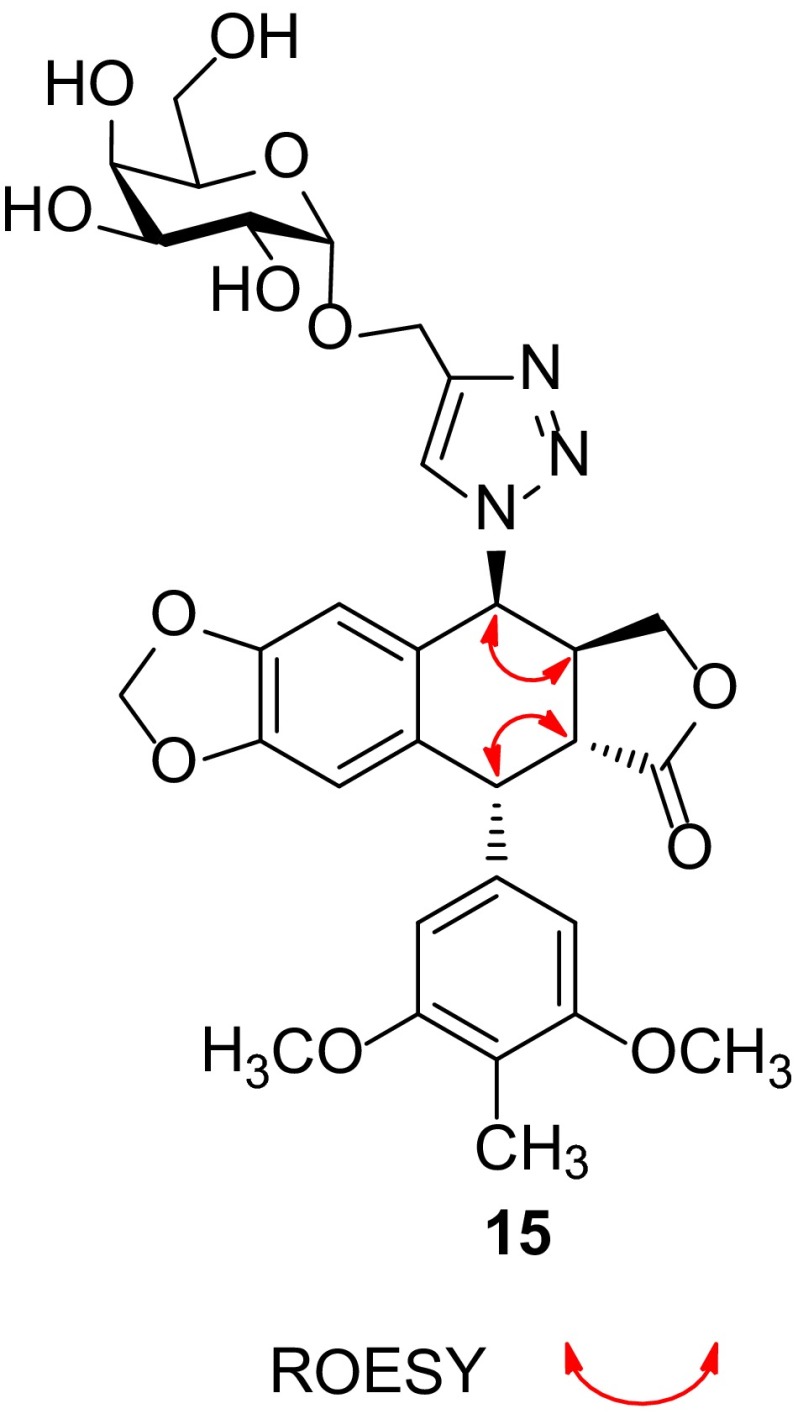
Key ROESY correlations in compound **15**

All the products were characterized by ^1^H-NMR, ^13^C-NMR, ESI-MS, and HRESI-MS. ESI-MS and HRESI-MS of all compounds showed the [M+Na]^+^ or [M+H]^+^ adduct as the molecular ion. In the ^1^H-NMR spectra, the formation of the triazole ring was confirmed by the resonance of its C^5*″*^–H signal (*δ* 7.81–8.31 ppm) in the aromatic region. The structure was further confirmed by the ^13^C-NMR spectra, which showed the two characteristic carbon signals at around 145 ppm (*δ*_C-4*″*_) and 126 ppm (*δ*_C-5*″*_) corresponding to the triazole residue. The configuration at C-4 position for target compounds **15**–**26** was confirmed based on the *J*_3,4_ coupling constant, which is typically < 5.0 Hz for 4*β*-substituted compounds due to a *cis* relationship between H-3 and H-4 [23]. In some cases, 4*β*-substitution was further confirmed by 2D-NMR spectral data. For example, the ROESY of compound **15** shows strong correlation between H-4 ↔ H-3 (Fig. 2), indicating that the *N*-linked triazole ring moiety is attached to C-4 of podophyllotoxin via a *β*-linkage. The coupling constant of the anomeric proton of d-galactose and d-xylose residues (*J*_1*′′′*,2*′′′*_) is typically < 5.0 Hz for the *α*-glycoside linkage. However, the coupling constant of the anomeric proton for d-mannose is usually small for both *α*- and *β*-mannosides. The α*-*linkage in d-mannosides was confirmed by the carbon-proton coupling constant (^2^*J*_C–H_) of the anomeric carbon by acquiring the non-decoupled ^13^C NMR spectra. For example, the ^2^*J*_C–H_ is 167.9 Hz for the anomeric carbon of the d-mannose residue in compound **23**, which confirms that **23** is an *α*-mannoside since the ^2^*J*_C–H_ of the anomeric carbon for a *β*-mannoside is typically below 160 Hz [24].

### Evaluation of Biological Activity

All 4*β*-triazole-podophyllotoxin glycosides **15**–**26** were tested for their anticancer activity against five human cancer cell lines, including HL-60 (leukemia), SMMC-7721 (hepatoma), A-549 (lung cancer), MCF-7 (breast cancer), and SW480 (colon cancer). Etoposide **(2**) and cisplatin were taken as reference compounds. The screening procedure was based on the standard MTT method [25], and the results are reported in the terms of IC_50_ values (Table 1).

**Table 1:** In vitro anticancer activity (IC_50_, μM) of compounds **15**–**26**

Compounds	IC_50_ (μM)				
	HL-60	SMMC-7721	A-549	MCF-7	SW480
**15**	> 40	> 40	> 40	> 40	> 40
**16**	2.85	3.99	4.07	7.28	5.52
**17**	> 40	> 40	> 40	> 40	> 40
**18**	> 40	> 40	> 40	> 40	> 40
**19**	> 40	> 40	> 40	> 40	> 40
**20**	> 40	> 40	> 40	> 40	> 40
**21**	16.67	20.50	38.89	38.51	> 40
**22**	> 40	> 40	> 40	> 40	> 40
**23**	> 40	> 40	>40	> 40	> 40
**24**	> 40	> 40	> 40	> 40	> 40
**25**	10.29	18.62	26.11	> 40	> 40
**26**	> 40	> 40	> 40	> 40	> 40
Etoposide (**2**)	0.31	8.12	11.92	32.82	17.11
Cisplatin	1.17	6.43	9.24	15.86	13.42

As it can be seen in Table 1, most of these compounds show weak cytotoxicity (IC_50_ > 40 μM). However, compound **16** shows strong anticancer activity against all cancer cell lines tested, with IC_50_ values ranging from 2.85 to 7.28 μM, which is significantly more potent than the control drug etoposide against four of the five cancer cells. It is interesting to note that the 4′-*O*-methylated analog **15** (IC_50_ > 40 μM) is much less potent than **16**, indicating that this 4′-*O*-hydroxy group is perhaps important for the anticancer activity of glycosylated podophyllotoxin derivatives.

### Chemical Stability Investigation

**Fig. 3:**
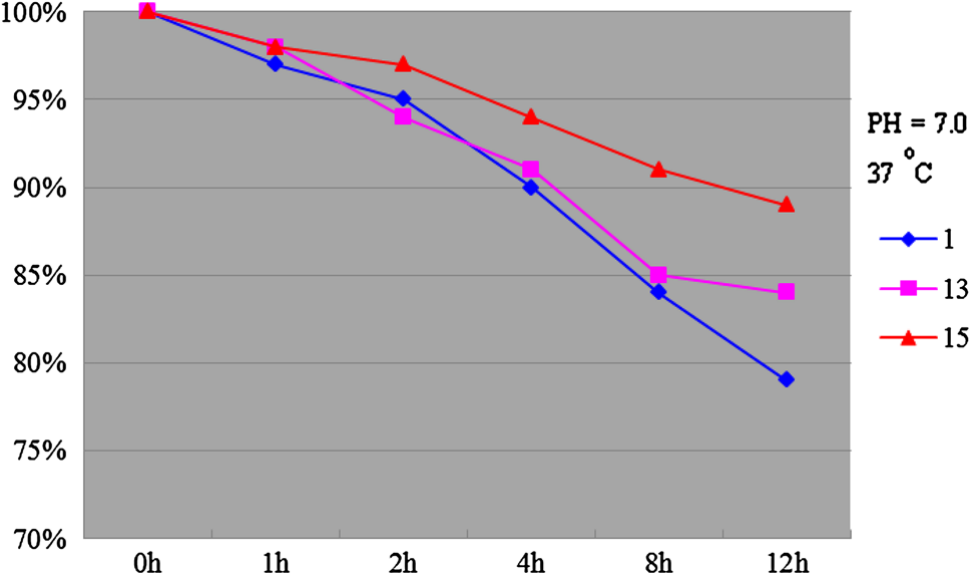
Chemical stability investigation of compounds **1**, **13** and **15**

Compound **15** was selected for the investigation of chemical stability in aqueous phase with comparison to podophyllotoxin (**1**) and 4*β*-azido-4-deoxypodophyllotoxin (**13**). The results indicated that compound **15** exhibits higher chemical stability under the physiological condition (37 °C, pH 7.0, Fig. 3) than both podophyllotoxin (**1**) and 4*β*-azido-4-deoxypodophyllotoxin (**13**). Hydrolysis of the* δ*-lactone is anticipated to be the main degradation pathway under this condition [26]. It appears that the incorporation of the d-galactose moiety slows down the hydrolysis and improves the chemical stability of the podophyllotoxin scaffold.

## Conclusions

In conclusion, we have used Fisher glycosylation strategy to prepare glycosylated terminal-alkynes. Then, all the glycosylated terminal-alkynes were reacted with podophyllotoxin-derived azides by the Click reaction to yield a series of 4*β*-triazole-podophyllotoxin glycosides **15**–**26** in good yields. All compounds were tested for anticancer activity against five human cancer cell lines. Most of these compounds show weak cytotoxicity while compound **16**, having a galactose residue directly linked to the triazole ring and a 4′-OH group on the E ring, is more potent than the anticancer drug etoposide against four of the five cancer cell lines tested. In addition, chemical stability investigation indicates that the conjugated sugar residue seems to improve the stability of the podophyllotoxin scaffold under the physiological condition.

## Experimental

### General

d-Galactose, and d-xylose were purchased from Sinopharm Chemical Reagent Co., Ltd (Shanghai, China). d-Mannose was purchased from Acros Organics (New Jersey, USA). Podophyllotoxin was obtained from Shanghai yuanye Bio-Technology Co., Ltd (Shanghai, China). Melting points were uncorrected. MS data were obtained in the ESI mode on API Qstar Pulsar instrument. HRMS data were obtained in the ESI mode on LCMS-IT-TOF (Shimadzu, Kyoto, Japan). NMR spectra were acquired on Bruker AV-400 or DRX-500 or Bruker AVANCE Ш-600 (Bruker BioSpin GmbH, Rheinstetten, Germany) instruments, using tetramethylsilane (TMS) as an internal standard. Column chromatography (CC) was performed on flash silica gel (200–300 mesh; Qingdao Makall Group Co., Ltd; Qingdao; China). All reactions were monitored using thin-layer chromatography (TLC) on silica gel plates.

### 2-[2-[2-(2-Propyn-1-yloxy)ethoxy]ethoxy-*α-*d-xylopyranoside (**12**)

d-Xylose (150 mg, 1 mmol) and *2*-*[2*-*(2*-*propargyloxyethoxy)ethoxy]ethanol***7** (940 mg, 5 mmol) were stirred at 65 °C. H_2_SO_4_–silica (5 mg) was added and stirring was continued until all solids had dissolved (~2.5 h). After cooling to room temperature, the reaction mixture was purified by CC on silica gel (CHCl_3_:CH_3_OH = 9:1) to afford **12** (96 mg, 30 %). ^1^H-NMR (CD_3_OD, 400 MHz) *δ* 4.77 (d, 1H, *J* = 3.7 Hz, C^1^–H), 4.19 (d, 2H, *J* = 2.3 Hz, CH_2_–C≡C), 3.83–3.78 (m, 1H), 3.72–3.69 (m, 2H), 3.67–3.66 (m, 6H), 3.55–3.44 (m, 4H), 3.36–3.33 (m, 2H), 2.87 (t, 1H, *J* = 2.4 Hz, C≡CH); ^13^C-NMR (CD_3_OD, 100 MHz) *δ* 100.6 (C-1), 80.7 (C≡CH), 76.1 (C≡CH), 75.3, 73.7, 71.6, 71.5, 71.4, 70.1, 68.2, 63.1 (C-6), 59.1 (CH_2_–C≡C); ESIMS: *m/z* 343 [M+Na]^+^, HRESIMS: calcd for C_14_H_24_O_18_Na [M+Na]^+^ 343.1363, found 343.1364.

### Click Chemistry: General Procedure for the Synthesis of Compounds **15**–**26**

To a solution of a terminal-alkyne **7**–**12** (0.2 mmol) and 4*β*-azido-podophyllotoxin analogues **13** or **14** (0.2 mmol) in *t*– BuOH–H_2_O (1:2, 1.0 mL) at room temperature were added copper (II) sulfate pentahydrate (0.02 mmol) and sodium ascorbate (1.0 M in H_2_O, 3 drops). The reaction mixture was stirred at room temperature for 4 h until the starting material disappeared as indicated by TLC. Then, the mixture was diluted with water (10 mL) and extracted with ethyl acetate (3 × 10 mL), and the combined organic layer was dried over sodium sulfate. The solvent was evaporated and the residue was purified by CC (CHCl_3_/CH_3_OH, 9:1) to afford the cycloaddition product **15**–**26** (75–87 %).

#### 4*β*-[4-(*α*-d-Galactopyranosyloxymethyl)-1,2,3-triazol-1-yl]-4-deoxypodophyllotoxin (**15**)

White amorphous powder, yield 86 %; mp 153–155 °C; [α]_D_^25.7^ +18.6 (*c* 0.28, CH_3_OH); ^1^H-NMR (CD_3_OD, 400 MHz) *δ* 7.84 (s, 1H, C^5*′′*^–H), 6.69 (s, 1H, C^5^–H), 6.62 (s, 1H, C^8^–H), 6.41 (s, 2H, C^2*′*^, C^6*′*^–H), 6.25 (d, 1H, *J* = 4.8 Hz, C^4^–H), 5.98–5.96 (m, 2H, OCH_2_O), 4.93 (d, 1H, *J* = 4.0 Hz, C^1*′′′*^–H), 4.83 (s, 1H, C^2*′′′*^–H), 4.80–4.78 (m, 1H), 4.64–4.61 (m, 1H), 4.69 (t, 1H, *J* = 8.0 Hz), 3.87–3.86 (m, 2H), 3.83–3.78 (m, 5H), 3.74 (s, 6H, C^3*′*^, C^5*′*^-OCH_3_), 3.72 (s, 3H, C^4*′*^-OCH_3_), 3.43 (dd, 1H, *J* = 5.0 Hz, 10.0 Hz, C^2*′′′*^–H), 3.20–3.15 (m, 1H, C^3^–H,); ^13^C-NMR (CD_3_OD, 100 MHz) *δ* 175.9 (C-12), 153.9 (C-3′, C-5′), 150.6 (C-7), 149.3 (C-6), 145.7 (C-4′′), 138.3 (C-1′), 136.8 (C-9), 134.8 (C-10), 126.9 (C-4′), 126.0 (C-5′′), 111.2 (C-5), 109.9 (C-8), 109.4 (C-2′, C-6′), 103.3 (OCH_2_O), 100.0 (C-1′′′), 72.8, 71.4, 71.1, 70.1, 68.9 (C-11), 62.9 (C-6′′), 61.5 (C-6′′′), 61.1 (4′-OCH_3_), 59.8 (C-4), 56.6 (3′,5′-OCH_3_), 44.9 (C-1), 42.5 (C-2), 38.6 (C-3); ESIMS: *m/z* 680 [M+Na]^+^, HRESIMS: calcd for C_31_H_35_N_3_O_13_Na [M+Na]^+^ 680.2062, found 680.2056.

#### 4*β*-[4-(*α*-d-Galactopyranosyloxymethyl)-1,2,3-triazol-1-yl]-4-deoxy-4′-demethylpodophyllotoxin (**16**)

White amorphous powder, yield 88 %; mp 162 °C; [α]_D_^25.6^ –9.8 (*c* 0.10, CH_3_OH); ^1^H-NMR (CD_3_OD, 400 MHz) *δ* 8.23 (s, 1H, C^5*′′*^–H), 6.62 (s, 1H, C^5^–H), 6.59 (s, 2H, C^2*′*^, C^6*′*^–H), 6.27 (s, 1H, C^8^–H), 6.04–5.93 (m, 3H, C^4^–H, OCH_2_O), 4.98 (d, 1H, *J* = 4.0 Hz, C^1*′′′*^–H), 4.72–4.68 (m, 2H), 4.23–4.19 (m, 2H), 3.90–3.87 (m, 3H), 3.80 (s, 6H, C^3*′*^, C^5*′*^–OCH_3_), 3.77–3.70 (m, 5H), 3.54–3.48 (m, 1H, C^3^–H); ^13^C-NMR (CD_3_OD, 125 MHz): *δ* 176.1 (C-12), 149.8 (C-7), 149.2 (C-6), 148.8 (C-3′, C-5′), 145.9 (C-4′′), 134.3 (C-9), 131.7 (C-10), 129.9 (C-1′), 129.2 (C-4′), 125.7 (C-5′′), 111.0 (C-5), 109.6 (C-2′, C-6′), 107.3 (C-8), 103.1 (OCH_2_O), 100.2 (C-1′′′), 72.8, 71.5, 71.3 (C-11), 71.2, 70.2, 64.1 (C-4), 62.9 (C-6′′), 61.8 (C-6′′′), 57.0 (3′, 5′-OCH_3_), 46.8 (C-1), 45.2 (C-2), 40.0 (C-3); ESIMS: *m/z* 666 [M+Na]^+^, HRESIMS: calcd for C_30_H_33_N_3_O_13_Na [M+Na]^+^ 666.1906, found 666.1900.

#### 4*β*-[4-(*α*-d-Mannopyranosyloxymethyl)-1,2,3-triazol-1-yl]-4-deoxypodophyllotoxin (**17**)

White amorphous powder, yield 83 %; mp 117–119 °C; [α]_D_^25.7^ –8.8 (*c* 0.21, CH_3_OH); ^1^H-NMR (C_5_D_5_N, 500 MHz) *δ* 8.31 (s, 1H, C^5*′′*^–H), 6.85 (s, 1H, C^5^–H), 6.82 (s, 1H, C^8^–H), 6.76 (s, 2H, C^2*′*^, C^6*′*^–H), 6.58 (d, 1H, *J* = 4.8 Hz, C^4^–H), 5.99–5.98 (m, 2H, OCH_2_O), 5.63 (s, 1H, C^1*′′′*^–H), 5.27 (d, 1H, *J* = 5.0 Hz, C^1^–H), 5.05–5.02 (m, 3H), 4.62–4.58 (m, 2H), 4.54–4.52 (m, 2H), 4.41–4.35 (m, 3H), 3.98–3.93 (m, 1H, C^2^–H), 3.85 (s, 6H, C^3*′*^, C^5*′*^–OCH_3_), 3.82 (s, 3H, C^4*′*^–OCH_3_), 3.46–3.40 (m, 1H, C^3^–H); ^13^C-NMR (C_5_D_5_N, 125 MHz) *δ* 174.1 (C-12), 153.5 (C-3′, C-5′), 149.4 (C-7), 147.8 (C-6), 145.1 (C-4′′), 140.1 (C-1′), 138.3 (C-9), 134.0 (C-10), 126.6 (C-5′′), 125.2 (C-4′), 110.7 (C-5), 109.5 (C-8), 109.2 (C-2′, C-6′), 102.4 (OCH_2_O), 101.7 (C-1′′′), 75.8, 72.9, 71.9, 69.1, 68.0 (C-11), 63.2 (C-6′′), 61.1 (C-6′′′), 60.6 (4′-OCH_3_), 58.8 (C-4), 56.3 (3′, 5′-OCH_3_), 44.3 (C-1), 41.9 (C-2), 38.0 (C-3); ESIMS: *m/z* 680 [M+Na]^+^, HRESIMS: calcd for C_31_H_35_N_3_O_13_H [M+H]^+^ 658.2243, found 658.2236.

#### 4*β*-[4-(*α*-d-Mannopyranosyloxymethyl)-1,2,3-triazol-1-yl]-4-deoxy-4′-demethylpodophyllotoxin (**18**)

White amorphous powder, yield 80 %; mp 208 °C; [α]_D_^26.7^ –72.8 (*c* 0.27, Pyridine); ^1^H-NMR (C_5_D_5_N, 400 MHz) *δ* 8.30 (s, 1H, C^5*′′*^–H), 6.84 (s, 2H, C^5^–H, C^8^–H), 6.80 (s, 2H, C^2*′*^, C^6*′*^–H), 6.52 (d, 1H, *J* = 4.8 Hz, C^4^–H), 5.93 (s, 2H, OCH_2_O), 5.62 (s, 1H, C^1*′′′*^–H), 5.24 (d, 1H, *J* = 5.0 Hz, C^1^–H), 5.03–5.00 (m, 3H), 4.64–4.57 (m, 2H), 4.53–4.51 (m, 2H), 4.42–4.36 (m, 3H), 3.89 (dd, 1H, *J* = 5.0 Hz, 10.0 Hz, C^2^–H), 3.72 (s, 6H, C^3*′*^, C^5*′′*^–OCH_3_), 3.43–3.38 (m, 1H, C^3^–H); ^13^C-NMR (C_5_D_5_N, 100 MHz) *δ* 174.2 (C-12), 149.4 (C-7), 148.7 (C-3′, C-5′), 148.1 (C-6), 145.1 (C-4′′), 137.4 (C-1′), 134.5 (C-9), 130.1 (C-10), 126.5 (C-5′′), 125.1 (C-4′), 110.7 (C-5), 109.7 (C-2′, C-6′), 109.3 (C-8), 102.3 (OCH_2_O), 101.9 (C-1′′′), 75.8, 72.9, 72.0, 69.2, 67.9 (C-11), 63.2 (C-6′′), 61.1 (C-6′′′), 58.8 (C-4), 56.5 (3′, 5′-OCH_3_), 44.1 (C-1), 42.0 (C-2), 37.9 (C-3); ESIMS: *m/z* 666 [M+Na]^+^, HRESIMS: calcd for C_30_H_33_N_3_O_13_H [M+H]^+^ 644.2086, found 644.2079.

#### 4*β*-[4-(*α*-d-Xylopyranosyloxymethyl)-1,2,3-triazol-1-yl]-4-deoxypodophyllotoxin (**19**)

White amorphous powder, yield 75 %; mp 125–128 °C; [α]_D_^25.7^ –35.9 (*c* 0.27, Pyridine); ^1^H-NMR (C_5_D_5_N, 500 MHz) *δ* 8.26 (s, 1H, C^5*′′*^–H), 6.81 (s, 1H, C^5^–H), 6.79 (s, 1H, C^8^–H), 6.75 (s, 2H, C^2*′*^, C^6*′*^–H), 6.53 (d, 1H, *J* = 5.0 Hz, C^4^–H), 5.96 (s, 2H, OCH_2_O), 5.42 (d, 1H, *J* = 2.9 Hz, C^1*′′′*^–H), 5.18 (d, 1H, *J* = 4.5 Hz, C^1^–H), 5.02–4.97 (m, 2H), 4.47–4.43 (m, 1H), 4.40–4.36 (m, 1H), 4.20–4.15 (m, 3H), 4.10–4.08 (m, 1H), 4.05–4.03 (m, 1H), 3.82 (s, 6H, C^3*′*^, C^5*′*^–OCH_3_), 3.80 (s, 3H, C^4*′*^–OCH_3_), 3.66–3.60 (m, 1H, C^2^–H), 3.40–3.38 (m, 1H, C^3^–H); ^13^C-NMR (C_5_D_5_N, 125 MHz) *δ* 174.1 (C-12), 153.5 (C-3′, C-5′), 148.3 (C-7), 147.7 (C-6), 145.4 (C-4′′), 140.0 (C-1′), 138.3 (C-9), 134.0 (C-10), 126.6 (C-5′′), 125.1 (C-4′), 110.7 (C-5), 109.4 (C-8), 109.2 (C-2′, C-6′), 102.4 (OCH_2_O), 100.5 (C-1′′′), 75.4, 73.7, 71.7, 68.0 (C-11), 63.8 (C-5′′′), 61.7 (C-6′′), 60.6 (4′-OCH_3_), 58.7 (C-4), 56.2 (3′, 5′-OCH_3_), 44.2 (C-1), 41.9 (C-2), 38.1 (C-3); ESIMS: *m/z* 650 [M+Na]^+^, HRESIMS: calcd for C_30_H_33_N_3_O_12_H [M+H]^+^ 628.2137, found 628.2132.

#### 4*β*-[4-(*α*-d-Xylopyranosyloxymethyl)-1,2,3-triazol-1-yl]-4-deoxy-4′-demethylpodophyllotoxin (**20**)

White amorphous powder, yield 84 %; mp 205–206 °C; [α]_D_^26.6^ –50.1 (*c* 0.14, Pyridine); ^1^H-NMR (C_5_D_5_N, 500 MHz) *δ* 8.24 (s, 1H, C^5*′′*^–H), 6.82 (s, 1H, C^5^–H), 6.78 (s, 2H, C^2*′*^, C^6*′*^–H), 6.76 (s, 1H, C^8^–H), 6.50 (d, 1H, *J* = 4.0 Hz, C^4^–H), 5.94–5.93 (m, 2H, OCH_2_O), 5.43 (d, 1H, *J* = 5.0 Hz, C^1*′′′*^–H), 5.20–5.18 (m, 1H), 5.02–5.00 (m, 1H), 4.96 (d, 1H, *J* = 5.0 Hz, C^1^–H), 4.47 (t, 1H, *J* = 8.0 Hz), 4.39 (t, 1H, *J* = 8.0 Hz), 4.20–4.17 (m, 3H), 4.10–4.08 (m, 1H), 4.06–4.04 (m, 1H), 3.73 (s, 6H, C^3*′*^, C^5*′*^–OCH_3_), 3.68–3.62 (m, 1H, C^2^–H), 3.42–3.39 (m, 1H, C^3^–H); ^13^C-NMR (C_5_D_5_N, 125 MHz) *δ* 174.2 (C-12), 149.4 (C-7), 148.8 (C-3′, C-5′), 148.2 (C-6), 145.4 (C-4′′), 137.4 (C-1′), 134.4 (C-9), 130.1 (C-10), 126.4 (C-5′′), 125.1 (C-4′), 110.8 (C-5), 109.7 (C-2′, C-6′), 109.3 (C-8), 102.4 (OCH_2_O), 100.5 (C-1′′′), 75.4, 73.8, 71.7, 68.0 (C-11), 63.8 (C-5′′′), 61.7 (C-6′′), 58.8 (C-4), 56.5 (3′, 5′-OCH_3_), 44.1 (C-1), 42.1 (C-2), 38.0 (C-3); ESIMS: *m/z* 636[M+Na]^+^, HRESIMS: calcd for C_29_H_31_N_3_O_12_H [M+H]^+^ 614.1980, found 614.1973.

#### 4*β*-{4-[1-(*α*-d-Galactopyranosyloxymethyl)-3,6,9-trioxadec-10-yl]-1,2,3-triazol-1-yl}-4-deoxypodophyllotoxin (**21**)

White amorphous powder, yield 82 %; mp 90 °C; [α]_D_^25.8^ +1.4 (*c* 0.17, CH_3_OH); ^1^H-NMR (CD_3_OD, 400 MHz) *δ* 7.82 (s, 1H, C^5*′′*^–H), 6.68 (s, 1H, C^5^–H), 6.62 (s, 1H, C^8^–H), 6.40 (s, 2H, C^2*′*^, C^6*′*^–H), 6.25 (d, 1H, *J* = 4.8 Hz, C^4^–H), 5.97–5.95 (m, 2H, OCH_2_O), 4.85 (s, 1H, C^1*′′′*^–H), 4.79 (d, 1H, *J* = 5.0 Hz, C^1^–H), 4.63–4.61 (m, 2H), 4.39 (t, 1H, *J* = 8.0 Hz), 3.87–3.80 (m, 7H), 3.73 (s, 6H, C^3*′*^, C^5*′*^–OCH_3_), 3.71 (s, 3H, C^4*′*^–OCH_3_), 3.69–3.60 (m, 12H, 3 × OCH_2_CH_2_O), 3.44 (dd, 1H, *J* = 5.0 Hz, 10.0 Hz, C^2^–H), 3.17–3.12 (m, 1H, C^3^–H); ^13^C-NMR (CD_3_OD, 125 MHz) *δ* 175.9 (C-12), 153.9 (C-3′, C-5′), 150.6 (C-7), 149.3 (C-6), 146.0 (C-4′′), 138.3 (C-1′), 136.8 (C-9), 134.8 (C-10), 127.0 (C-4′), 126.0 (C-5′′), 111.2 (C-5), 109.8 (C-8), 109.4 (C-2′, C-6′), 103.3 (OCH_2_O), 100.6 (C-1′′′), 72.4, 71.6, 71.4, 71.3, 71.1, 70.9, 70.4, 68.9 (C-11), 68.1, 65.0 (C-6′′), 62.8 (C-6′′′), 61.1 (4′-OCH_3_), 59.8 (C-4), 56.6 (3′, 5′-OCH_3_), 44.9 (C-1), 42.5 (C-2), 38.6 (C-3); ESIMS: *m/z* 812 [M+Na]^+^, HRESIMS: calcd for C_37_H_47_N_3_O_16_H [M+H]^+^ 790.3029, found 790.3013.

#### 4*β*-{4-[1-(*α*-d-Galactopyranosyloxymethyl)-3,6,9-trioxadec-10-yl]-1,2,3-triazol-1-yl}-4-deoxy-4′-demethylpodophyllotoxin (**22**)

White amorphous powder, yield 87 %; mp 128 °C; [α]_D_^25.6^ –0.3 (*c* 0.16, CH_3_OH); ^1^H-NMR (CD_3_OD, 400 MHz) *δ* 7.82 (s, 1H, C^5*′′*^–H), 6.68 (s, 1H, C^5^–H), 6.64 (s, 1H, C^8^–H), 6.37 (s, 2H, C^2*′*^, C^6*′*^–H), 6.25 (d, 1H, *J* = 4.8 Hz, C^4^–H), 5.98–5.96 (m, 2H, OCH_2_O), 4.85 (s, 1H, C^1*′′′*^–H), 4.77 (d, 1H, *J* = 5.0 Hz, C^1^–H), 4.68–4.67 (m, 2H), 4.39 (t, 1H, *J* = 8.0 Hz), 3.87–3.83 (m, 7H), 3.74 (s, 6H, C^3*′′*^, C^5*′′*^–OCH_3_), 3.70–3.61 (m, 12H, 3 × OCH_2_CH_2_O), 3.41 (dd, 1H, *J* = 5.0 Hz, 10.0 Hz, C^2^–H), 3.17–3.12 (m, 1H, C^3^–H); ^13^C-NMR (CD_3_OD, 100 MHz) *δ* 176.0 (C-12), 150.5 (C-7), 149.2 (C-6), 148.7 (C-3′, C-5′), 145.9 (C-4′′), 136.0 (C-1′), 135.1 (C-9), 131.3 (C-10), 126.9 (C-4′), 126.0 (C-5′′), 111.2 (C-5), 109.7 (C-8), 109.3 (C-2′, C-6′), 103.2 (OCH_2_O), 100.6 (C-1′′′), 72.4, 71.6, 71.4, 71.3, 71.1, 70.9, 70.4, 68.9 (C-11), 68.1, 65.0 (C-6′′), 62.8 (C-6′′′), 59.9 (C-4), 56.7 (3′, 5′-OCH_3_), 44.7 (C-1), 42.7 (C-2), 38.5 (C-3); ESIMS: *m/z* 798 [M+Na]^+^, HRESIMS: calcd for C_36_H_45_N_3_O_16_H [M+H]^+^ 776.2873, found 776.2861.

#### 4*β*-{4-[1-(*α*-d-Mannopyranosyloxymethyl)-3,6,9-trioxadec-10-yl]-1,2,3-triazol-1-yl}-4-deoxypodophyllotoxin (**23**)

White amorphous powder, yield 81 %; mp 94 °C; [α]_D_^26.8^ –13.1 (*c* 0.20, CH_3_OH); ^1^H-NMR (CD_3_OD, 400 MHz) *δ* 7.82 (s, 1H, C^5*′′*^–H), 6.67 (s, 1H, C^5^–H), 6.63 (s, 1H, C^8^–H), 6.40 (s, 2H, C^2*′*^, C^6*′*^–H), 6.25 (d, 1H, *J* = 4.8 Hz, C^4^–H), 5.97–5.95 (m, 2H, OCH_2_O), 4.78 (s, 2H, C^1*′′′*^–H, C^1^–H), 4.64–4.61 (m, 2H), 4.40–4.36 (m, 1H), 3.84-3.79 (m, 7H), 3.73 (s, 6H, C^3*′*^, C^5*′*^–OCH_3_), 3.71 (s, 3H, C^4*′*^–OCH_3_), 3.65–3.59 (m, 12H, 3 × OCH_2_CH_2_O), 3.43 (dd, 1H, *J* = 5.0 Hz, 10.0 Hz, C^2^–H), 3.17–3.12 (m, 1H, C^3^–H); ^13^C-NMR (CD_3_OD, 100 MHz) *δ* 175.9 (C-12), 154.0 (C-3′, C-5′), 150.6 (C-7), 149.3 (C-6), 146.0 (C-4′′), 138.3 (C-1′), 136.8 (C-9), 134.8 (C-10), 127.0 (C-4′), 126.0 (C-5′′), 111.2 (C-5), 109.9 (C-8), 109.4 (C-2′, C-6′), 103.3 (OCH_2_O), 101.7 (C-1′′′), 74.6, 72.5, 72.1, 71.6, 71.5, 71.3, 70.9, 68.9 (C-11), 68.6, 67.7, 65.0 (C-6′′), 63.0 (C-6′′′), 61.1 (4′-OCH_3_), 59.8 (C-4), 56.6 (3′, 5′-OCH_3_), 44.9 (C-1), 42.5 (C-2), 38.6 (C-3); ESIMS: *m/z* 812 [M+Na]^+^, HRESIMS: calcd for C_37_H_47_N_3_O_16_H [M+H]^+^ 790.3029, found 790.3012.

#### 4*β*-{4-[1-(*α*-d-Mannopyranosyloxymethyl)-3,6,9-trioxadec-10-yl]-1,2,3-triazol-1-yl}-4-deoxy-4′-demethylpodophyllotoxin (**24**)

White amorphous powder, yield 78 %; mp 108 °C; [α]_D_^26.9^ –26.2 (*c* 0.14, CH_3_OH); ^1^H-NMR (CD_3_OD, 400 MHz) *δ* 7.81 (s, 1H, C^5*′′*^–H), 6.68 (s, 1H, C^5^–H), 6.64 (s, 1H, C^8^–H), 6.38 (s, 2H, C^2*′*^, C^6*′*^–H), 6.25 (d, 1H, *J* = 4.8 Hz, C^4^–H), 5.98–5.97 (m, 2H, OCH_2_O), 4.78 (s, 2H, C^1*′′′*^–H), 4.77 (d, 1H, *J* = 5.0 Hz, C^1^–H), 4.64–4.62 (m, 2H), 4.41–4.37 (m, 1H), 3.84–3.79 (m, 7H), 3.75 (s, 6H, C^3*′*^, C^5*′*^–OCH_3_), 3.68–3.58 (m, 12H, 3 × OCH_2_CH_2_O), 3.41 (dd, 1H, *J* = 5.0 Hz, 10.0 Hz, C^2^–H), 3.18–3.13 (m, 1H, C^3^–H); ^13^C-NMR (CD_3_OD, 100 MHz) *δ* 176.0 (C-12), 150.5 (C-7), 149.2 (C-6), 148.7 (C-3′, C-5′), 146.0 (C-4′′), 136.0 (C-1′), 135.1 (C-9), 131.3 (C-10), 126.9 (C-4′), 125.9 (C-5′′), 111.2 (C-5), 109.7 (C-8), 109.3 (C-2′, C-6′), 103.2 (OCH_2_O), 101.7 (C-1′′′), 74.6, 72.5, 72.1, 71.6, 71.5, 71.3, 70.9, 68.9 (C-11), 68.6, 67.7, 65.0 (C-6′′), 63.0 (C-6′′′), 59.9 (C-4), 56.8 (3′, 5′-OCH_3_), 44.7 (C-1), 42.7 (C-2), 38.5 (C-3); ESIMS: *m/z* 798 [M+Na]^+^, HRESIMS: calcd for C_36_H_45_N_3_O_16_H [M+H]^+^ 776.2873, found 776.2863.

#### 4*β*-{4-[1-(*α*-d-Xylopyranosyloxymethyl)-3,6,9-trioxadec-10-yl]-1,2,3-triazol-1-yl}-4-deoxypodophyllotoxin (**25**)

White amorphous powder, yield 80 %; mp 113–115 °C; [α]_D_^26.5^ –8.3 (*c* 0.18, CH_3_OH); ^1^H-NMR (CD_3_OD, 400 MHz) *δ* 7.82 (s, 1H, C^5*′′*^–H), 6.68 (s, 1H, C^5^–H), 6.60 (s, 1H, C^8^–H), 6.41 (s, 2H, C^2*′*^, C^6*′*^–H), 6.25 (d, 1H, *J* = 4.8 Hz, C^4^–H), 5.97–5.95 (m, 2H, OCH_2_O), 4.78 (d, 1H, *J* = 5.2 Hz, C^1^–H), 4.75 (d, 1H, *J* = 4.0 Hz, C^1*′′′*^–H), 4.64–4.61 (m, 2H), 4.39–4.36 (m, 1H), 3.85–3.75 (m, 2H), 3.73 (s, 6H, C^3*′*^, C^5*′*^–OCH_3_), 3.71 (s, 3H, C^4*′*^–OCH_3_), 3.67–3.58 (m, 12H, 3 × OCH_2_CH_2_O), 3.52–3.40 (m, 4H), 3.36–3.32 (m, 1H, C^2^–H), 3.17–3.15 (m, 1H, C^3^–H); ^13^C-NMR (CD_3_OD, 100 MHz) *δ* 175.8 (C-12), 154.0 (C-3′, C-5′), 150.6 (C-7), 149.3 (C-6), 146.0 (C-4′′), 138.3 (C-1′), 136.8 (C-9), 134.8 (C-10), 127.0 (C-4′), 126.0 (C-5′′), 111.2 (C-5), 109.9 (C-8), 109.4 (C-2′, C-6′), 103.3 (OCH_2_O), 100.6 (C-1′′′), 75.3, 73.7, 71.5, 71.5, 71.3, 71.3, 70.9, 68.9 (C-11), 68.2, 65.0 (C-5′′′), 63.1 (C-6′′), 61.1 (4′-OCH_3_), 59.8 (C-4), 56.6 (3′, 5′-OCH_3_), 44.9 (C-1), 42.5 (C-2), 38.6 (C-3); ESIMS: *m/z* 782 [M+Na]^+^, HRESIMS: calcd for C_36_H_45_N_3_O_15_H [M+H]^+^ 760.2923, found 760.2914.

#### 4*β*-{4-[1-(*α*-d-Xylopyranosyloxymethyl)-3,6,9-trioxadec-10-yl]-1,2,3-triazol-1-yl}-4-deoxy-4′-demethylpodophyllotoxin (**26**)

White amorphous powder, yield 84 %; mp 103 °C; [α]_D_^26.5^ –9.7 (*c* 0.27, CH_3_OH); ^1^H-NMR (CD_3_OD, 400 MHz) *δ* 7.83 (s, 1H, C^5*′′*^–H), 6.70 (s, 1H, C^5^–H), 6.66 (s, 1H, C^8^–H), 6.40 (s, 2H, C^2*′*^, C^6*′*^–H), 6.28 (d, 1H, *J* = 4.8 Hz, C^4^–H), 6.00–5.98 (m, 2H, OCH_2_O), 4.79 (d, 1H, *J* = 4.0 Hz, C^1*′′′*^–H), 4.77 (d, 1H, *J* = 3.6 Hz, C^1^–H), 4.66–4.64 (m, 2H), 4.41 (t, 1H, *J* = 12.0 Hz), 3.77 (s, 6H, C^3*′*^, C^5*′*^–OCH_3_), 3.70–3.62 (m, 12H, 3 × OCH_2_CH_2_O), 3.55–3.53 (m, 4H), 3.48–3.40 (m, 2H), 3.38–3.35 (m, 1H, C^2^–H), 3.17–3.15 (m, 1H, C^3^–H); ^13^C-NMR (CD_3_OD, 100 MHz) *δ* 176.4 (C-12), 150.9 (C-7, C-6), 149.7 (C-4′′), 149.1 (C-3′, C-5′), 136.4 (C-1′), 135.5 (C-9), 131.8 (C-10), 127.3 (C-4′), 126.4 (C-5′′), 111.7 (C-5), 110.2 (C-8), 109.8 (C-2′, C-6′), 103.7 (OCH_2_O), 110.0 (C-1′′′), 75.3, 73.7, 71.5, 71.4, 71.4, 71.3, 70.9, 68.9 (C-11), 68.2, 65.0 (C-5′′′), 63.1 (C-6′′), 60.3 (C-4), 57.2 (3′, 5′-OCH_3_), 45.2 (C-1), 43.1 (C-2), 39.0 (C-3); ESIMS: *m/z* 768 [M+Na]^+^, HRESIMS: calcd for C_35_H_43_N_3_O_15_H [M+H]^+^ 746.2767, found 746.2755.

### Cell Culture and Cytotoxicity Assay

The following human tumor cell lines were used: HL-60, SMMC-7721, A-549, MCF-7, and SW480. All the cells were cultured in RMPI-1640 or DMEM medium (Hyclone, Logan, UT, USA), supplemented with 10 % fetal bovine serum (Hyclone) at 37 °C in a humidified atmosphere with 5 % CO_2_. Cell viability was assessed by conducting colorimetric measurements of the amount of insoluble formazan formed in living cells based on the reduction of 3-(4,5-dimethyl-thiazol-2-yl)-2,5-diphenyltetrazolium bromide (MTT) (Sigma, St. Louis, MO, USA). Briefly, adherent cells (100 μL) were seeded into each well of a 96-well cell culture plate and allowed to adhere for 12 h before drug addition, while suspended cells were seeded just before drug addition, both with an initial density of 1 × 10^5^ cells/mL in 100 μL of medium. Each tumor cell line was exposed to the test compound at various concentrations in triplicate for 48 h. After the incubation, MTT (100 μg) was added to each well, and the incubation continued for 4 h at 37 °C. The cells lysed with SDS (200 μL) after removal of 100 μL of medium. The optical density of lysate was measured at 595 nm in a 96-well microtiter plate reader (Bio-Rad 680). The IC_50_ value of each compound was calculated by Reed and Muench’s method [25].
